# Supplementary material for: Delta neutrophil index for predicting mortality in trauma patients who underwent emergent abdominal surgery: A case controlled study
Source: PLoS One. 2020 Mar 23;15(3):e0230149. doi: 10.1371/journal.pone.0230149 (PMC7089524; doi:10.1371/journal.pone.0230149)
Supplement: S1 Table — (DOCX) [file pone.0230149.s001.docx]

S1 Table. Comparison of other variables between survivors and non-survivors.

|  | Survivor (n=150) | Non-survivor (n=19) | Total  (n=169) | p-value |
| --- | --- | --- | --- | --- |
| Associated injury |  |  |  |  |
| Head&neck (AIS ≥3) | 9(6.0%) | 3(15.8%) | 12(7.1%) | 0.138* |
| Face (AIS ≥3) | 1(0.7%) | 1(5.3%) | 2(1.2%) | 0.213* |
| Chest (AIS ≥3) | 33(22.0%) | 9(47.4%) | 42(24.9%) | 0.016 |
| Extremities&pelvis (AIS ≥3) | 9(6.0%) | 7(36.8%) | 16(9.5%) | <0.001 |
| ER CPR | 1 (0.7%) | 6 (31.6%) | 7 (4.1%) | <0.001 |
| Damage control surgery | 4 (2.7%) | 11 (73.3%) | 15 (8.9%) | <0.001 |
| Shock on ICU admission | 12 (8.0%) | 18 (94.7%) | 30 (17.8%) | <0.001 |
| Serum Albumin level (mg/dL) |  |  |  |  |
| Initial | 3.6±0.7 | 3.0±0.9 | 3.5±0.8 | 0.001 |
| Postoperative | 3.2±0.5 | 2.2±0.7 | 3.1±0.6 | <0.001 |
| POD1 | 3.1±0.6 | 2.7±0.5 | 3.1±0.6 | 0.005 |
| POD2 | 3.0±0.5 | 2.2±1.3 | 2.9±0.7 | 0.045 |
| Operation time (min) | 121±58 | 147±93 | 124±63 | 0.250 |

AIS, abbreviated injury scale; ER, emergency room; CPR, cardiopulmonary resuscitation; ICU, intensive care unit; POD, postoperative day
