# Supplementary material for: Delta neutrophil index for predicting mortality in trauma patients who underwent emergent abdominal surgery: A case controlled study
Source: PLoS One. 2020 Mar 23;15(3):e0230149. doi: 10.1371/journal.pone.0230149 (PMC7089524; doi:10.1371/journal.pone.0230149)
Supplement: Attachment — Submitted filename: Response to Reviewers.docx [file pone.0230149.s004.docx]

PONE-D-19-35229R1

Delta neutrophil index for predicting mortality in trauma patients who underwent emergent abdominal surgery: A case controlled study

PLOS ONE

Dear Dr. Jang,

I am returning the revised manuscript since I need some clarifications.

You respond to each of the first reviewer's questions but except in one case I do not see in your response letter a description of what changes did you make within the manuscript. Associated to this issue, when I examined the marked copy, I did not always understand why certain places were highlighted.

I suggest the following. Under each of the reviewer's questions introduce two subheadings: authors' response, desciption of changes made. Thus for each of the questions we will have your answer followed by an explanation if changes were made in the manuscript and their location. If no changes were made, just write that no changes were made.

Thank you,

Itamar Ashkenazi M.D.

Academic Editor

Dear Academic Editor

As you mentioned, we answered reviewer’s questions point-by-point and clearly stated whether the manuscript was corrected or not. Also we highlighted again in the only changed points of the manuscript.

Thank you for your comments.

Ji Young Jang M.D.

Review Comments to the Author

Please use the space provided to explain your answers to the questions above. You may also include additional comments for the author, including concerns about dual publication, research ethics, or publication ethics. (Please upload your review as an attachment if it exceeds 20,000 characters)

Reviewer #1: The article by Jang explores mortality prediction in trauma patients undergoing emergent abdominal surgery.

The DNI is the immature granulocyte fraction determined by subtracting the fraction of mature PMN leukocytes from the sum of myeloperoxidase-reactive cells and reflects the number of immature neutrophils. It has been used in sepsis and several other conditions.

Several questions: 1. What were the inclusion/exclusion criteria?

🡪 To clarify the inclusion and exclusion criteria

We added a patient flow chart as Figure 1(line 87-88).

2. Was the study exploring in hospital deaths only or was there an attempt to examine 28 days mortality or longer? How does the death distribution look like?

🡪 Thirteen (68.4%) patients died within 7 days, and 5 (26.3%) died between 7 and 28 days. The other one died of sepsis after 28 days.

We added this contents to the Results section (line 140-141).

3. In Table 1&3, is this the mean?

🡪 As we described in ‘statistical analysis’ section of patients and methods, continuous variables were mainly presented as the mean±standard deviation. When no following a normal distribution, it was expressed as median(range).

4. Can a predictor based on DNI difference and the other idependent variable identified and POD be presented in an aggregate score so that it would have clinical utility for the practicing surgeon?

🡪 In multivariated analysis, POD1 DNI, postoperative lactate, and postoperative SOFA score were identified as independent risk factors related to mortality. Authors confirmed the AUC using the ROC curve of these variables, and confirmed that the cut-off value of POD1 DNI was the criterion having the highest sensitivity and specificity at 7.1%. The postoperative lactate and SOFA scores were also analyzed in the same way, indicating cut-off levels of 5.1 and 6.5.

Indeed, in our hospital, it was possible to recognize the high probability of death in patients with elevated DNI levels above 7.1% the day after surgery. DNI helped surgeons explain the patient’s condition early to the caregiver and determine further evaluation and general ward transfer. However, we did not calculate a score that combines these three scores.

Authors added this content to the discussion section (line 232-235).

5. Could the author add a sentence about the scientific rationale why increased DNI is associated with mortality?

🡪 DNI can be seen as a value representing the proportion of immature granulocytes. As SIRS caused by tissue damage increases the release of damage-associated molecular patterns (DAMPs), SIRS becomes more severe and the multiple organ failure of the patient worsens. In addition, when neutrophils are functionally impaired by SIRS, the release of immature banded neutrophils increases to compensate for this, which is indicated by an increase in DNI.

Since this content is already described in the discussion section, authors did not make any additional descriptions (239-248^th^ line).

Reviewer #2: The manuscript looks interesting, well written and intelligible.

The methodology of the study looks appropriate and rigorous.

The work seems to add something new to the field of trauma research.

My recommendation is to accept.Review Comments to the Author

Please use the space provided to explain your answers to the questions above. You may also include additional comments for the author, including concerns about dual publication, research ethics, or publication ethics. (Please upload your review as an attachment if it exceeds 20,000 characters)

Reviewer #1: The article by Jang explores mortality prediction in trauma patients undergoing emergent abdominal surgery.
The DNI is the immature granulocyte fraction determined by subtracting the fraction of mature PMN leukocytes from the sum of myeloperoxidase-reactive cells and reflects the number of immature neutrophils. It has been used in sepsis and several other conditions.
Several questions: 1. What were the inclusion/exclusion criteria?
2. Was the study exploring in hospital deaths only or was there an attempt to examine 28 days mortality or longer? How does the death distribution look like?
3. In Table 1&3, is this the mean?
4. Can a predictor based on DNI difference and the other idependent variable identified and POD be presented in an aggregate score so that it would have clinical utility for the practicing surgeon?
5. Could the author add a sentence about the scientific rationale why increased DNI is associated with mortality?

Reviewer #2: The manuscript looks interesting, well written and intelligible.
The methodology of the study looks appropriate and rigorous.
The work seems to add something new to the field of trauma research.
My recommendation is to accept.PONE-D-19-35229
Delta neutrophil index for predicting mortality in trauma patients who underwent emergent abdominal surgery: A case controlled study
PLOS ONE

Dear Dr. Jang,

Thank you for submitting your manuscript to PLOS ONE. After careful consideration, we feel that it has merit but does not fully meet PLOS ONE’s publication criteria as it currently stands. Therefore, we invite you to submit a revised version of the manuscript that addresses the points raised during the review process.

We would appreciate receiving your revised manuscript by Mar 26 2020 11:59PM. When you are ready to submit your revision, log on to https://www.editorialmanager.com/pone/ and select the 'Submissions Needing Revision' folder to locate your manuscript file.

If you would like to make changes to your financial disclosure, please include your updated statement in your cover letter.

To enhance the reproducibility of your results, we recommend that if applicable you deposit your laboratory protocols in protocols.io, where a protocol can be assigned its own identifier (DOI) such that it can be cited independently in the future. For instructions see: <http://journals.plos.org/plosone/s/submission-guidelines#loc-laboratory-protocols>

Please include the following items when submitting your revised manuscript:

- A rebuttal letter that responds to each point raised by the academic editor and reviewer(s). This letter should be uploaded as separate file and labeled 'Response to Reviewers'.
- A marked-up copy of your manuscript that highlights changes made to the original version. This file should be uploaded as separate file and labeled 'Revised Manuscript with Track Changes'.
- An unmarked version of your revised paper without tracked changes. This file should be uploaded as separate file and labeled 'Manuscript'.

Please note while forming your response, if your article is accepted, you may have the opportunity to make the peer review history publicly available. The record will include editor decision letters (with reviews) and your responses to reviewer comments. If eligible, we will contact you to opt in or out.

We look forward to receiving your revised manuscript.

Kind regards,

Itamar Ashkenazi
Academic Editor
PLOS ONE

PONE-D-19-35229
Delta neutrophil index for predicting mortality in trauma patients who underwent emergent abdominal surgery: A case controlled study
PLOS ONE

Dear Dr. Jang,

Thank you for submitting your manuscript to PLOS ONE. After careful consideration, we feel that it has merit but does not fully meet PLOS ONE’s publication criteria as it currently stands. Therefore, we invite you to submit a revised version of the manuscript that addresses the points raised during the review process.

We would appreciate receiving your revised manuscript by Mar 26 2020 11:59PM. When you are ready to submit your revision, log on to https://www.editorialmanager.com/pone/ and select the 'Submissions Needing Revision' folder to locate your manuscript file.

If you would like to make changes to your financial disclosure, please include your updated statement in your cover letter.

To enhance the reproducibility of your results, we recommend that if applicable you deposit your laboratory protocols in protocols.io, where a protocol can be assigned its own identifier (DOI) such that it can be cited independently in the future. For instructions see: <http://journals.plos.org/plosone/s/submission-guidelines#loc-laboratory-protocols>

Please include the following items when submitting your revised manuscript:

- A rebuttal letter that responds to each point raised by the academic editor and reviewer(s). This letter should be uploaded as separate file and labeled 'Response to Reviewers'.
- A marked-up copy of your manuscript that highlights changes made to the original version. This file should be uploaded as separate file and labeled 'Revised Manuscript with Track Changes'.
- An unmarked version of your revised paper without tracked changes. This file should be uploaded as separate file and labeled 'Manuscript'.

Please note while forming your response, if your article is accepted, you may have the opportunity to make the peer review history publicly available. The record will include editor decision letters (with reviews) and your responses to reviewer comments. If eligible, we will contact you to opt in or out.

We look forward to receiving your revised manuscript.

Kind regards,

Itamar Ashkenazi
Academic Editor
PLOS ONE

PONE-D-19-35229
Delta neutrophil index for predicting mortality in trauma patients who underwent emergent abdominal surgery: A case controlled study
PLOS ONE

Dear Dr. Jang,

Thank you for submitting your manuscript to PLOS ONE. After careful consideration, we feel that it has merit but does not fully meet PLOS ONE’s publication criteria as it currently stands. Therefore, we invite you to submit a revised version of the manuscript that addresses the points raised during the review process.

We would appreciate receiving your revised manuscript by Mar 26 2020 11:59PM. When you are ready to submit your revision, log on to https://www.editorialmanager.com/pone/ and select the 'Submissions Needing Revision' folder to locate your manuscript file.

If you would like to make changes to your financial disclosure, please include your updated statement in your cover letter.

To enhance the reproducibility of your results, we recommend that if applicable you deposit your laboratory protocols in protocols.io, where a protocol can be assigned its own identifier (DOI) such that it can be cited independently in the future. For instructions see: <http://journals.plos.org/plosone/s/submission-guidelines#loc-laboratory-protocols>

Please include the following items when submitting your revised manuscript:

- A rebuttal letter that responds to each point raised by the academic editor and reviewer(s). This letter should be uploaded as separate file and labeled 'Response to Reviewers'.
- A marked-up copy of your manuscript that highlights changes made to the original version. This file should be uploaded as separate file and labeled 'Revised Manuscript with Track Changes'.
- An unmarked version of your revised paper without tracked changes. This file should be uploaded as separate file and labeled 'Manuscript'.

Please note while forming your response, if your article is accepted, you may have the opportunity to make the peer review history publicly available. The record will include editor decision letters (with reviews) and your responses to reviewer comments. If eligible, we will contact you to opt in or out.

We look forward to receiving your revised manuscript.

Kind regards,

Itamar Ashkenazi
Academic Editor
PLOS ONE

PONE-D-19-35229
Delta neutrophil index for predicting mortality in trauma patients who underwent emergent abdominal surgery: A case controlled study
PLOS ONE

Dear Dr. Jang,

Thank you for submitting your manuscript to PLOS ONE. After careful consideration, we feel that it has merit but does not fully meet PLOS ONE’s publication criteria as it currently stands. Therefore, we invite you to submit a revised version of the manuscript that addresses the points raised during the review process.

We would appreciate receiving your revised manuscript by Mar 26 2020 11:59PM. When you are ready to submit your revision, log on to https://www.editorialmanager.com/pone/ and select the 'Submissions Needing Revision' folder to locate your manuscript file.

If you would like to make changes to your financial disclosure, please include your updated statement in your cover letter.

To enhance the reproducibility of your results, we recommend that if applicable you deposit your laboratory protocols in protocols.io, where a protocol can be assigned its own identifier (DOI) such that it can be cited independently in the future. For instructions see: <http://journals.plos.org/plosone/s/submission-guidelines#loc-laboratory-protocols>

Please include the following items when submitting your revised manuscript:

- A rebuttal letter that responds to each point raised by the academic editor and reviewer(s). This letter should be uploaded as separate file and labeled 'Response to Reviewers'.
- A marked-up copy of your manuscript that highlights changes made to the original version. This file should be uploaded as separate file and labeled 'Revised Manuscript with Track Changes'.
- An unmarked version of your revised paper without tracked changes. This file should be uploaded as separate file and labeled 'Manuscript'.

Please note while forming your response, if your article is accepted, you may have the opportunity to make the peer review history publicly available. The record will include editor decision letters (with reviews) and your responses to reviewer comments. If eligible, we will contact you to opt in or out.

We look forward to receiving your revised manuscript.

Kind regards,

Itamar Ashkenazi
Academic Editor
PLOS ONE

PONE-D-19-35229
Delta neutrophil index for predicting mortality in trauma patients who underwent emergent abdominal surgery: A case controlled study
PLOS ONE

Dear Dr. Jang,

Thank you for submitting your manuscript to PLOS ONE. After careful consideration, we feel that it has merit but does not fully meet PLOS ONE’s publication criteria as it currently stands. Therefore, we invite you to submit a revised version of the manuscript that addresses the points raised during the review process.

We would appreciate receiving your revised manuscript by Mar 26 2020 11:59PM. When you are ready to submit your revision, log on to https://www.editorialmanager.com/pone/ and select the 'Submissions Needing Revision' folder to locate your manuscript file.

If you would like to make changes to your financial disclosure, please include your updated statement in your cover letter.

To enhance the reproducibility of your results, we recommend that if applicable you deposit your laboratory protocols in protocols.io, where a protocol can be assigned its own identifier (DOI) such that it can be cited independently in the future. For instructions see: <http://journals.plos.org/plosone/s/submission-guidelines#loc-laboratory-protocols>

Please include the following items when submitting your revised manuscript:

- A rebuttal letter that responds to each point raised by the academic editor and reviewer(s). This letter should be uploaded as separate file and labeled 'Response to Reviewers'.
- A marked-up copy of your manuscript that highlights changes made to the original version. This file should be uploaded as separate file and labeled 'Revised Manuscript with Track Changes'.
- An unmarked version of your revised paper without tracked changes. This file should be uploaded as separate file and labeled 'Manuscript'.

Please note while forming your response, if your article is accepted, you may have the opportunity to make the peer review history publicly available. The record will include editor decision letters (with reviews) and your responses to reviewer comments. If eligible, we will contact you to opt in or out.

We look forward to receiving your revised manuscript.

Kind regards,

Itamar Ashkenazi
Academic Editor
PLOS ONE

PONE-D-19-35229
Delta neutrophil index for predicting mortality in trauma patients who underwent emergent abdominal surgery: A case controlled study
PLOS ONE

Dear Dr. Jang,

Thank you for submitting your manuscript to PLOS ONE. After careful consideration, we feel that it has merit but does not fully meet PLOS ONE’s publication criteria as it currently stands. Therefore, we invite you to submit a revised version of the manuscript that addresses the points raised during the review process.

We would appreciate receiving your revised manuscript by Mar 26 2020 11:59PM. When you are ready to submit your revision, log on to https://www.editorialmanager.com/pone/ and select the 'Submissions Needing Revision' folder to locate your manuscript file.

If you would like to make changes to your financial disclosure, please include your updated statement in your cover letter.

To enhance the reproducibility of your results, we recommend that if applicable you deposit your laboratory protocols in protocols.io, where a protocol can be assigned its own identifier (DOI) such that it can be cited independently in the future. For instructions see: <http://journals.plos.org/plosone/s/submission-guidelines#loc-laboratory-protocols>

Please include the following items when submitting your revised manuscript:

- A rebuttal letter that responds to each point raised by the academic editor and reviewer(s). This letter should be uploaded as separate file and labeled 'Response to Reviewers'.
- A marked-up copy of your manuscript that highlights changes made to the original version. This file should be uploaded as separate file and labeled 'Revised Manuscript with Track Changes'.
- An unmarked version of your revised paper without tracked changes. This file should be uploaded as separate file and labeled 'Manuscript'.

Please note while forming your response, if your article is accepted, you may have the opportunity to make the peer review history publicly available. The record will include editor decision letters (with reviews) and your responses to reviewer comments. If eligible, we will contact you to opt in or out.

We look forward to receiving your revised manuscript.

Kind regards,

Itamar Ashkenazi
Academic Editor
PLOS ONE

Journal Requirements:
