# Supplementary material for: Delta neutrophil index for predicting mortality in trauma patients who underwent emergent abdominal surgery: A case controlled study
Source: PLoS One. 2020 Mar 23;15(3):e0230149. doi: 10.1371/journal.pone.0230149 (PMC7089524; doi:10.1371/journal.pone.0230149)
Supplement: Attachment — Submitted filename: Response to reviewers 2.docx [file pone.0230149.s005.docx]

PONE-D-19-35229R2

Delta neutrophil index for predicting mortality in trauma patients who underwent emergent abdominal surgery: A case controlled study

PLOS ONE

Dear Dr. Jang,

Thank you for submitting your manuscript to PLOS ONE. Thank you also for clarifying the reviewers' comments and making the appropriate changes. I want to aplogize but there is one more issue that needs to be dealt with before considerning this manuscript suitable for publication.

ISS and SOFA are scores based on several components. Considering these as continuous variables is not appropriate. There is no ISS 15 for example. ISS 16 is not twice as bad as ISS 8...

I recommend either presenting these as medians and ranges, or presenting proportion of patients above a certain threshold. For example, proportion of patients with ISS of 16 or higher.

Please introduce these before I hand in the final decision.

- Thank you for your important comment.

The authors discussed the reviewer’s opinion and decided to present proportion (ISS>15) and median (range) for the ISS. However, the SOFA score on ICU admission was linearly correlated with the mortality rate of patients, we decided to keep it as a continuous variable. Since the standard deviation of SOFA score is relatively large, we modified it to median (range).

- The multivariate analysis was re-run using the modified ISS>15 and confirmed no change in the main results. In addition, tables and manuscript were revised.

Reference

*Ferreira et al. Serial evaluation of the SOFA score to predict outcome in critically ill patients. JAMA.2001 Oct 10;286(14):1754-8*

Thank you gain.

Ji Young Jang M.D.
